# The effects of rutin supplement on blood pressure markers, some serum antioxidant enzymes, and quality of life in patients with type 2 diabetes mellitus compared with placebo

**DOI:** 10.3389/fnut.2023.1214420

**Published:** 2023-08-03

**Authors:** Hadi Bazyar, Ahmad Zare Javid, Akram Ahangarpour, Ferdows Zaman, Seyed Ahmad Hosseini, Vida Zohoori, Vahideh Aghamohammadi, Shima Yazdanfar, Mohammad Ghasemi Deh Cheshmeh

**Affiliations:** ^1^Student Research Committee, Ahvaz Jundishapur University of Medical Sciences, Ahvaz, Iran; ^2^Department of Public Health, Sirjan School of Medical Sciences, Sirjan, Iran; ^3^Student Research Committee, Sirjan School of Medical Sciences, Sirjan, Iran; ^4^Nutrition and Metabolic Diseases Research Center, Clinical Sciences Research Institute, Ahvaz Jundishapur University of Medical Sciences, Ahvaz, Iran; ^5^Department of Nutrition, School of Allied Medical Sciences, Ahvaz Jundishapur University of Medical Sciences, Ahvaz, Iran; ^6^Department of Physiology, Faculty of Medicine, Ahvaz Jundishapur University of Medical Sciences, Ahvaz, Iran; ^7^Health Research Institute, Diabetes Research Center, Ahvaz Jundishapur University of Medical Sciences, Ahvaz, Iran; ^8^Teesside University, Middlesbrough, United Kingdom; ^9^Department of Nutrition, Khalkhal University of Medical Science, Khalkhal, Iran

**Keywords:** rutin, type 2 diabetes mellitus, quality of life, blood pressure, antioxidant enzymes

## Abstract

**Background:**

This trial aimed to investigate the effects of rutin supplement in type 2 diabetes mellitus (T2DM) patients.

**Methods:**

In this trial with a double-blind and controlled design, fifty patients were randomly divided into intervention (*n* = 25) and control groups (*n* = 25) and were treated with 1 g of rutin or placebo for three months, respectively. At the baseline and end of the intervention, mean arterial pressure (MAP), heart rate (HR), pulse pressure (PP), systolic and diastolic blood pressure (SBP and DBP), serum levels of antioxidant enzymes, such as catalase (CAT), glutathione peroxidase (GPx), and superoxide dismutase (SOD) and quality of life (QOL) parameters, were evaluated.

**Results:**

Rutin consumption caused a significant reduction in SBP, DBP, PP, MAP, and HR, with a significant increase in SOD, CAT, and GPx and some QOL parameters (emotional limitations, energy and freshness, mental health, social performance, and general health) compared with baseline (*p* for all <0.05). Also, the mean changes of emotional limitations, energy and freshness, mental health, and general health (unadjusted *p* for all <0.05) and GPX and SOD (adjusted *p* for all <0.05) were significantly higher in the rutin group compared with the placebo group. Although, in the supplement group compared with the placebo group, the mean changes of SBP, DBP, MAP, PP, and HR were significantly lower (adjusted *p* for all <0.05).

**Conclusion:**

Rutin consumption improved blood pressure, the levels of antioxidant enzymes, and QOL in patients with T2DM.

## Introduction

Diabetes mellitus (DM) is one of the fastest-growing chronic diseases worldwide that is caused by pancreatic beta-cell dysfunction (or a decrease in the number of β-cells) or insulin resistance and is associated with hyperglycemia (1). In the last three decades, the number of diabetic patients has quadrupled. According to global statistics, the prevalence of diabetes is reported in 1 in 11 adults, 90% of whom suffer from type 2 diabetes mellitus (T2DM) (2, 3). In Iran, the prevalence of T2DM in the adult population is estimated at 11.4% (4). The pathogenesis of diabetes is complex, and various genetic and environmental factors are involved in it (5). Numerous changes in intracellular metabolic pathways caused by chronic hyperglycemia and dyslipidemia are involved in damage to vital tissues and organs of the body, such as the heart, kidneys, blood vessels, and nerves, and diabetes complications (6). So, in addition to controlling hyperglycemia, management of obesity, hypertension, dyslipidemia, and platelet activation are also effective in treating this disease (7). Hyperglycemia and activation of the nuclear factor-κB (NF-κB) pathway stimulate the production of reactive oxygen species (ROS), which lead to the induction of insulin resistance and subsequently reduce the activity of antioxidant enzymes including superoxide dismutase (SOD), glutathione peroxidase (GPx), and catalase (CAT) (8). On the other hand, oxidative stress, which occurs with an imbalance between the antioxidant defense system and ROS production, is considered an important risk factor in developing and progressing diabetes complications, such as hypertension and its mortality (9, 10). Also, it has been shown in diabetic patients, quality of life (QOL) was associated with factors, such as hyperglycemia, diabetes duration, insulin therapy, sex, age, complications of diabetes-induced oxidative stress, and other comorbidities (11). Diabetic patients with high levels of blood glucose have a lower QOL compared with healthy people (12). Controlling oxidative stress and complications of diabetes can be effective in increasing the QOL of diabetic patients (13). Nutrition therapy and dietary supplements as adjunct therapy can play an important role in the management of diabetes, improving inflammation, and the QOL in patients with DM (14, 15). So, the protective role of flavonoids as powerful antioxidants is known in oxidative stress-related chronic diseases, such as diabetes (16).

Rutin or 3,3′,4′,5,7-pentahydroxyflavone-3-rhamnoglucoside is a natural flavonoid glycoside that is produced as one of the common secondary metabolites of plants and is also known as rutoside, sophorin, vitamin P, or quercetin-3-O-rutinoside (17). Buckwheat, green tea, citrus, apples, and grapes are food sources of rutin (18). The antioxidant, anti-diabetic, anti-hypertensive, cardio-protective, and anti-inflammatory properties of rutin have been reported in studies (19, 20). The ability of rutin to reduce ROS production and increase the levels of different antioxidants in cells has also been reported. The results of a study in rats showed that rutin increased the activity of endogenous hepatic antioxidant enzymes, such as GPx, CAT, SOD, glutathione-S-transferase (GST), glutathione reductase (GSR), and glutathione (GSH) (21). Therefore, rutin is known as an anti-inflammatory flavonoid that has pharmacological activities such as lowering blood pressure, increase blood flow, and maintaining elasticity and strengthening capillaries (22). Also, a human study showed that the use of rutin along with vitamin C played an important role in increasing the QOL of diabetic patients (23). We hypothesized that rutin improves blood pressure, antioxidant status, and QOL in T2DM patients. According to our review, the present study was the first well-designed clinical trial to evaluate the effects of rutin in diabetic patients. So, this 3-month intervention was designed to determine the effects of rutin supplement on blood pressure markers, some serum antioxidant enzymes, and QOL in patients with T2DM compared with placebo.

## Materials and methods

### Study design and participants

**Figure 1:**
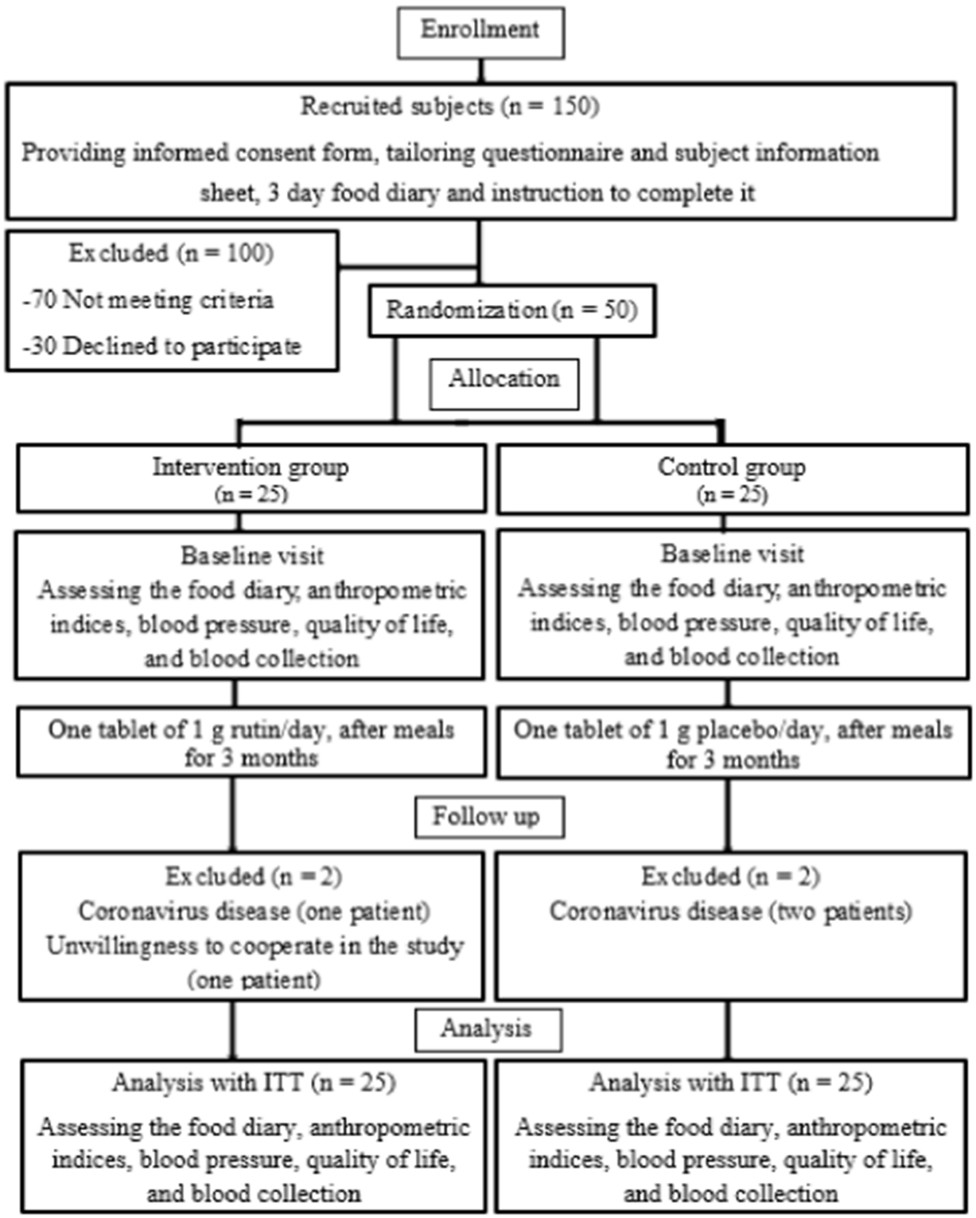
Stages of clinical trial progress.

This trial was done as a double-blind, randomized, placebo-controlled interventional study at the Endocrinology and Metabolism clinic of Golestan Hospital, Ahvaz Jundishapur University of Medical Sciences, Iran, between May 2021 and October 2021. Out of 150 diabetic patients, 50 patients were selected (based on inclusion criteria) and were randomly divided into intervention (*n* = 25) and control groups (*n* = 25). Another 100 patients were excluded from the study for not meeting the inclusion criteria inclusion criteria (*n* = 70) and unwillingness to participate (*n* = 30) (Figure 1). The following criteria were used to diagnose diabetes: 2-h glucose (2 hpp) ≥ 200 mg/dL or fasting blood glucose (FBG) ≥126 mg/dL, or glycosylated hemoglobin (HbA1c) ≥ 6.5% (24).

### Sample size

Considering systolic blood pressure (SBP) as the main variable and based on the study of Zahedi et al. (25), a sample size of 19 people was calculated for each group (confidence interval = 95% and power = 90%, $n=\frac{{\left(z_{1}-\frac{\alpha }{2}+z_{1}-\beta \right)}^{2}\left(\delta_{1}{}^{2}+\delta_{2}{}^{2}\right)}{{\left(\mu_{1}-\mu_{2}\right)}^{2}}$). Also, using a 25% drop, 5 people were added to each group, and the final sample size was 24 people for each group. In this study, the primary outcome was SBP and other factors, including parameters of blood pressure, antioxidant enzymes, and QOL parameters, were considered as the secondary outcome.

### Ethical considerations

The informed consent form was completed by all volunteers participating before starting the supplementation and patients were informed about the details of the study. The protocol of this clinical trial was confirmed by the Ethics Committee of the Ahvaz Jundishapur University of Medical Science (IR.AJUMS.REC.1400.110). Moreover, this study was registered on the Iranian Registry of Clinical Trials website (IRCT ID: IRCT20170116031993N5).

### Inclusion and exclusion criteria

The inclusion of patients in the study was done according to the following criteria: male and female, age range 18 to 60 years, disease period 2 to 15 years, HbA1c between 6.5 and 11% according to other studies (26), and body mass index (BMI) less than 35 kg/m^2^. Exclusion criteria included people with kidney or endocrine diseases, insulin injection, consumption of antioxidants and anti-inflammatory drugs, anemia, smoking and alcohol consumption, significant weight loss or gain, pregnancy and lactation, and use of unusual diets.

### Randomization and blinding

In this study, randomization was performed using “Random Allocation Software” (RAS) with block randomization protocol (6 blocks with 4 codes). Therefore, 50 patients with T2DM were randomly allocated to two groups of intervention and control (25 patients in each group). Allocation concealment was performed using 2 codes, A and B, to reduce systematic error. To do this work, each can containing supplement or placebo tablets received a code A or B (coding was done by someone who was out of the study but had information about the research work). Also, to increase the accuracy of the work, supplement and placebo tablets (in terms of shape, taste, size, and color) and cans containing these tablets (in terms of color, size, and shape) were completely similar. In this trial, the researcher, patients, and physician (clinical consultant) were blinded to the supplementation. In addition, the person who performed the laboratory tests did not know the details of the study.

### Intervention procedure

Both intervention and control groups took medications prescribed by the physician (except insulin). The duration of treatment was three months. During this period, patients in the supplement group received one 1 g tablet of rutin daily after the meals (27). The Solgar company (USA) made the supplement tablets. Each 1 g tablet contained 500 mg of pure rutin and 500 mg of other ingredients (glycerin, magnesium stearic, plant cellulose, di-calcium phosphate, microcrystalline cellulose, stearic acid, and silica). Also, patients in the control group received one 1 g tablet of placebo daily after the meals. Placebo tablets were prepared by the Faculty of Pharmacy at Ahvaz Jundishapur University of Medical Sciences (Iran) and each 1 g placebo tablet contained the same components as the supplement (glycerin, magnesium stearic, plant cellulose, di-calcium phosphate, microcrystalline cellulose, stearic acid, and silica) except for the pure rutin. Toxic effects have not been reported from rutin consumption at a dose of 500 mg (28).

### Following and compliance

Patients were asked not to change their diet during the intervention period, to take the medication prescribed by the physician (without changing the dose and number), and to maintain their levels of physical activity. The researcher monitored the use of tablets by the patients twice a month through phone call or text message. The compliance assessment was done according to the number of returned tablets. Patients who had taken less than 90% of the tablets were not evaluated for the second stage and were excluded from the study process. Also, the side effects of rutin consumption were assessed during these three months.

### Evaluation of anthropometric indices, dietary intake, and physical activity

A nutritionist measured all anthropometric indices. A Seca digital scale (the model number: 786, made in Germany) was used to assess weight and height. Body mass was measured with a precision of 0.5 kg with the least clothing and without shoes. Height was also measured with a precision of 0.1 cm without shoes. Body mass index (BMI) was calculated using a person’s body mass and height (body mass in kilograms divided by height in meters squared) (29). Measurement of waist and hip circumference (WC and HC) was done using a tape measure with a precision of 0.5 cm. WC was measured as the widest part of the body between the edge of the lower rib and the upper iliac crest at the end of a normal exhalation. The most prominent hip area was considered to measure HC. Nutritionist 4 (NUT 4) software was used to assess the patients’ diets. So, the amount of energy intake, macronutrients, and micronutrients were calculated for each patient at the beginning and end of the study. The International Physical Activity Questionnaire (IPAQ) was considered to calculate the physical activity of the patients (30).

### Measurement of blood pressure

After 20 min of rest, blood pressure was measured between 8:00 and 9:00 in the morning by an experienced nurse and the average of three consecutive measurements was recorded. Systolic and diastolic blood pressure (SBP and DBP) and heart rate (HR) were calculated by the Omron digital sphygmomanometer (model M3, made in Vietnam). In addition, mean arterial pressure (MAP) and pulse pressure (PP) were obtained with the following formulas (31): PP(mmHg) = SBP (mmHg) – DBP (mmHg) and MAP(mmHg)=[SBP+(2×DBP)]/3. All instruments used in this study, such as the scale, tape measure, and sphygmomanometer, and sampling place were the same at the start and end of the study.

### QOL assessment

QOL questionnaire was used to assess QOL in patients with T2DM. This questionnaire had 36 items that evaluated eight different aspects of health, including physical performance (10 items), emotional limitations (3 items), physical limitations (4 items), mental health (5 items), energy and freshness (4 items), social performance (2 items), physical pain (2 items), and general health (6 items). The lowest score in each aspect was zero and the highest was 100. The validity and reliability of this questionnaire were calculated in Iran (*r* = 0.7–0.9) (32).

### Blood collection and biochemical tests

Blood samples (5 mL) were taken from diabetic patients before and after the end of the three-month treatment period (after 12 h of overnight fasting and before taking the medication). Blood samples were poured into anticoagulant tubes and serum was separated by centrifugation (3,000 g for 15 min). Serum samples were stored in the freezer at a temperature of −70°C to be used to assess the serum levels of antioxidant enzymes after the end of the intervention period and serum collection from all patients. Serum levels of antioxidant enzymes (SOD, CAT, and GPx) were measured using Zell Bio kits (GmbH, Germany) and the method of enzyme-linked immunosorbent assay (ELISA).

### Data analysis

Data were reported as mean ± standard deviation (SD) for quantitative data and frequency (percentage) for qualitative data. The Kolmogorov–Smirnov test was applied to examine the normal distribution in the two groups. A comparison of results before and after the supplementation within each group was performed using Paired *t*-test and if distribution was not normal, the Wilcoxon test was used. Also, for comparison of qualitative variables between the rutin and placebo groups, the Chi-square test was applied. At the beginning and the end of the intervention, the Independent *t*-test was used to compare quantitative variables between the two groups (supplement and placebo) and the Mann–Whitney test was used, if the data distribution was not normal. After the treatment and in the crude model, a comparison of mean changes (after – before) between the two groups was done based on the Independent *t*-test and if the data distribution was not normal, analysis of results was done using the Mann–Whitney test. In addition, in the adjusted model and after removing the confounders (BMI, WC, sex, age, race, education, job, medications, physical activity, disease duration, and energy), the Analysis of covariance (ANCOVA) test was used to compare the mean of changes between the intervention and control group. The ITT approach was applied to compensate for the withdrawal. The analysis of the results was done using SPSS software (version 23, Chicago, IL, USA). Significance was considered as a value of *p* less than 0.05.

## Results

Two patients in the rutin group were excluded due to coronavirus disease and unwillingness to continue and two patients in the control group were excluded because of coronavirus disease. Finally, four patients re-entered the analysis using ITT approach and results were analyzed in each group with 25 patients (Figure 1). Patients did not report any side effect from rutin consumption during the study.

### Baseline characteristics

According to Table 1, at the beginning of the intervention, there was no significant difference in sex, age, education, job, race, anthropometric indices (WC, HC, height, body mass, and BMI), medications (data not shown), physical activity, and disease duration between the two groups of rutin and placebo (*p* for all ≥0.05) (Table 1). Furthermore, diet analysis did not show a significant difference between the patients’ energy intake, macronutrients, including carbohydrate, protein, and fat, and micronutrients (beta-carotene, α-tocopherol, vitamin A, C, E, and selenium) at the beginning and end of the supplementation period (*p* for all ≥0.05) (Table 2).

**Table 1:** The characteristics of subjects at baseline.

Variables	Control group (*n* = 25)	Intervention group (*n* = 25)	^*^p-value
Gender (N) (%)			
Female	14 (56)	13 (52)	0.77^a^
Male	11 (44)	12 (48)	
Age (years)	52.40 ± 5.62	51.88 ± 7.07	0.77
Height (cm)	168.24 ± 10.23	169.24 ± 8.14	0.70
Weight (kg)	79.68 ± 7.52	81.20 ± 11.91	0.59
BMI (kg/m^2^)	28.34 ± 3.64	28.27 ± 2.95	0.94
WC (cm)	101.08 ± 7.10	102.44 ± 9.85	0.57
HC (cm)	103.72 ± 6.07	104.20 ± 7.61	0.80
Race (*N*) (%)			
Fars	6 (24)	5 (20)	0.69^a^
Lor	9 (36)	7 (28)	
Arab	10 (40)	13 (52)	
Education (*N*) (%)			
Illiterate – elementary	10 (40)	7 (28)	0.54^a^
Middle – school	5 (20)	7 (28)	
High – school	6 (24)	9 (36)	
College	4 (16)	2 (8)	
Job (*N*) (%)			
Unemployed	2 (8)	4 (16)	0.64^a^
Labor	5 (20)	7 (28)	
Housekeeper	13 (52)	11 (44)	
Employee	5 (20)	3 (12)	
Physical Activity (met-min/week)	310.36 ± 154.71	354.04 ± 144.70	0.30
Disease duration (years)	7.64 ± 3.06	7.16 ± 3.53	0.61

Values are expressed as means ± SD. *p* < 0.05 was considered as significant. *p < 0.05 was considered as significant using Independent *t*-test between the two groups at baseline. ^a^*p* < 0.05 was considered as significant using Chi-square test.

**Table 2:** Mean ± SD of energy, macronutrients, and micronutrients intake at baseline and post-intervention.

Variables	Baseline (*n* = 25)	Post-intervention (*n* = 25)	*p*-value**
Energy (kcal/d)			
Control group	1947.61 ± 181.10	1912.17 ± 131.71	0.17
Intervention group	2003.38 ± 159.95	1973.25 ± 176.89	0.18
*p*-value*	0.25	0.17	
Carbohydrate (g/d)			
Control group	252.95 ± 22.90	250.06 ± 16.54	0.35
Intervention group	260.87 ± 20.91	258.133 ± 23.32	0.35
*p*-value*	0.20	0.16	
Protein (g/d)			
Control group	76.79 ± 6.99	75.52 ± 4.82	0.21
Intervention group	79.37 ± 6.16	78.13 ± 6.90	0.15
*p*-value*	0.17	0.12	
Fat (g/d)			
Control group	68.61 ± 5.86	67.29 ± 4.11	0.11
Intervention group	70.76 ± 5.52	69.71 ± 6.38	0.16
*p*-value*	0.18	0.11	
Cholesterol (g/d)			
Control group	153.11 ± 34.13	152.26 ± 31.60	0.56
Intervention group	144.18 ± 39.52	145.23 ± 41.29	0.44
*p*-value*	0.39	0.50	
Vitamin A (mcg/d)			
Control group	373.41 ± 109.91	395.38 ± 83.53	0.21
Intervention group	375.89 ± 142.71	367.95 ± 102	0.77
*p*-value*	0.94	0.30	
Beta-Carotene (mcg/d)			
Control group	4371.53 ± 1631.69	4360.10 ± 1178.56	0.97
Intervention group	4434.78 ± 1738.14	4079.71 ± 1005.72	0.32
*p*-value*	0.89	0.37	
Selenium (mcg/d)			
Control group	47.99 ± 21.21	51.96 ± 16.51	0.46
Intervention group	58.08 ± 22.33	51.52 ± 22.71	0.27
*p*-value*	0.10	0.93	
Vitamin C (mg/d)			
Control group	99.00 ± 38.93	97.27 ± 29.88	0.81
Intervention group	87.33 ± 30.97	94.81 ± 33.01	0.40
*p*-value*	0.24	0.78	
α-tocopherol (mg/d)			
Control group	7.70 ± 1.68	7.51 ± 1.67	0.15
Intervention group	7.40 ± 1.95	7.60 ± 2.10	0.46
*p-*value*	0.56	0.86	
Vitamin E (mg/d)			
Control group	2.36 ± 0.70	2.18 ± 0.56	0.17
Intervention group	2.22 ± 0.69	2.17 ± 0.51	0.75
*p*-value*	0.47	0.91	

*p < 0.05 was considered as significant at baseline and significant post-intervention using Independent *T*-test between two groups. **p < 0.05 was considered as significant using Paired *T*-test (within group).

### Rutin and markers of blood pressure

Biochemical evaluation revealed that markers of blood pressure (SBP, MAP, DBP, HR, and PP) were not significantly different between the rutin and placebo groups at the baseline of the study (*p* ≥ 0.05). In response to treatment with rutin, the mean levels of DBP, SBP, MAP, HR, and PP decreased significantly compared with the baseline (86.48 ± 7.48 vs. 82.28 ± 8.24, *p* = 0.001; 129.44 ± 12.84 vs. 120.84 ± 10.89, *p* < 0.001; 100.80 ± 7.59 vs. 95.16 ± 7.49, *p* < 0.001; 88.40 ± 8.16 vs. 83.04 ± 9.70, *p* < 0.001; 42.96 ± 12.47 vs. 38.64 ± 11.37, *p* = 0.01; respectively). Also, in the supplement group compared with the placebo group, the mean changes of SBP, DBP, MAP, PP, and HR (−8.60 ± 9.32 vs. 1.16 ± 5.18, *p* < 0.001; −4.20 ± 5.78 vs. 0.48 ± 2.40, *p* = 0.001; −5.64 ± 6.02 vs. 0.70 ± 2.58, *p* < 0.001; −4.32 ± 8.27 vs. 0.88 ± 3.87, *p* = 0.007; −5.36 ± 5.67 vs. 0.48 ± 3.22, *p* < 0.001; respectively) were significantly lower. In addition, after the adjustment of confounding factors, the intervention with rutin showed a significant reduction in the mean changes of SBP, MAP, DBP, PP, and HR compared with the control group (*p* < 0.001, *p* < 0.001, *p* = 0.001, *p* = 0.01, and *p* < 0.001) (Table 3).

**Table 3:** Markers of blood pressure at baseline and post-intervention.

Variables	Intervention group (*n* = 25)	Control group (*n* = 25)	*p*-Value ^**^	*p*-Value***	*p*-Value****
SBP (mmHg)					
Baseline	129.44 ± 12.84	131.20 ± 17.15	0.68		
After 3 months	120.84 ± 10.89	132.36 ± 16.83	0.006		
*p*-value*	< 0.001	0.27			
Difference	−8.60 ± 9.32	1.16 ± 5.18		< 0.001	< 0.001
DBP (mmHg)					
Baseline	86.48 ± 7.48	85.92 ± 11.88	0.84		
After 3 months	82.28 ± 8.24	86.40 ± 11.07	0.14		
*p*-value*	0.001	0.32			
Difference	−4.20 ± 5.78	0.48 ± 2.40		0.001	0.001
MAP (mmHg)					
Baseline	100.80 ± 7.59	101.01 ± 13.00	0.94		
After 3 months	95.16 ± 7.49	101.72 ± 12.33	0.02		
*p*-value*	< 0.001	0.18			
Difference	−5.64 ± 6.02	0.70 ± 2.58		< 0.001	< 0.001
PP (mmHg)					
Baseline	42.96 ± 12.47	45.48 ± 10.17	0.43		
After 3 months	38.64 ± 11.37	46.36 ± 10.16	0.01		
*p*-value*	0.01	0.26			
Difference	−4.32 ± 8.27	0.88 ± 3.87		0.007	0.01
HR (plus/min)					
Baseline	88.40 ± 8.16	84.40 ± 8.74	0.10		
After 3 months	83.04 ± 9.70	84.88 ± 8.06	0.46		
*p*-value*	< 0.001	0.18			
Difference	−5.36 ± 5.67	0.48 ± 3.22		< 0.001	< 0.001

Values are expressed as means ± SD. *p < 0.05 was considered as significant using Paired *t*-test. **p < 0.05 was considered as significant using Independent *t*-test between the two groups at baseline and post-intervention. ***p < 0.05 was considered as significant difference using Independent *t*-test between the two groups post-intervention. ****p < 0.05 was considered as significant using Analysis of covariance (ANCOVA) between the two groups post-intervention after adjusting for confounding factors.

### Rutin and serum antioxidant enzymes

At the start of the study, the serum levels of CAT, GPX, and SOD did not significantly differ between the supplement and placebo groups (*p* ≥ 0.05). Intervention with the rutin for 3 months indicated a significant increase in the mean levels of CAT, GPX, and SOD compared with the baseline (21.16 ± 3.58 vs. 22.51 ± 3.41, *p* = 0.002; 255.36 ± 51.70 vs. 278.64 ± 41.15, *p* = 0.04; 27.31 ± 3.04 vs. 28.45 ± 2.76, *p* = 0.01; respectively). In crude model, the mean changes of GPX and SOD were significantly higher in the treatment group compared with the control group (23.28 ± 36.34 vs -3.72 ± 16.96, *p* = 0.002; 1.14 ± 2.03 vs –0.60 ± 1.93, *p* = 0.003; respectively). Also, after adjusting the confounders, the mean changes of GPX and SOD were significantly higher in the rutin group compared with the placebo group (*p* = 0.01 and *p* = 0.003, respectively). However, no significant difference was seen in the mean changes of CAT between the intervention and control groups in crude and adjusted model (*p* = 0.15 and *p* = 0.69, respectively) (Table 4).

**Table 4:** Serum antioxidant enzymes at baseline and post-intervention.

Variables	Intervention group (*n* = 25)	Control group (*n* = 25)	*p*-value^**^	*p*-value***	*p*-value****
SOD (U/mL)					
Baseline	27.31 ± 3.04	26.19 ± 2.95	0.19		
After 3 months	28.45 ± 2.76	25.58 ± 2.99	0.001		
*p*-value*	0.01	0.12			
Difference	1.14 ± 2.03	−0.60 ± 1.93		0.003	0.003
CAT (U/mL)					
Baseline	21.16 ± 3.58	22.16 ± 4.09	0.36		
After 3 months	22.51 ± 3.41	22.71 ± 3.75	0.84		
*p*-value*	0.002	0.15			
Difference	1.34 ± 1.97	0.55 ± 1.88		0.15	0.69
GPX (U/mL)					
Baseline	255.36 ± 51.70	231.36 ± 46.80	0.09		
After 3 months	278.64 ± 41.15	227.64 ± 42.81	< 0.001		
*p*-value*	0.004	0.28			
Difference	23.28 ± 36.34	−3.72 ± 16.96		0.002	0.01

Values are expressed as means ± SD. *p < 0.05 was considered as significant using Paired *t*-test. **p < 0.05 was considered as significant using Independent *t*-test between the two groups at baseline and post-intervention. ***p < 0.05 was considered as significant difference using Independent *t*-test between the two groups post-intervention. ****p < 0.05 was considered as significant using Analysis of covariance (ANCOVA) between the two groups post-intervention after adjusting for confounding factors.

### Rutin and QOL parameters

At the baseline, no significant difference was observed in the QOL parameters, including physical performance, physical limitations, emotional limitations, energy and freshness, mental health, social performance, physical pain, and general health, between the intervention and control groups (*p* ≥ 0.05). The 3-month treatment with rutin resulted in a significant increase in some QOL parameters, such as emotional limitations (40.64 ± 45.16 vs. 68.04 ± 35.38, *p* = 0.006), energy and freshness (50.60 ± 11.93 vs. 66.60 ± 12.88, *p* < 0.001), mental health (52.16 ± 14.02 vs. 61.96 ± 10.63, *p* < 0.001), social performance (67.56 ± 19.45 vs. 74.72 ± 12.17, *p* = 0.02), and general health (43.48 ± 10.46 vs. 61.48 ± 12.49, *p* < 0.001) compared with the initial value. Also, after taking the rutin supplement, a significant difference was observed in the mean changes of emotional limitations, energy and freshness, mental health, and general health between the two groups of supplement and placebo (27.40 ± 41.69 vs –8.64 ± 26.92, *p* = 0.002: 16.00 ± 8.66 vs –1.40 ± 3.95, *p* < 0.001: 9.80 ± 8.92 vs –1.12 ± 4.54, *p* < 0.001: 18.00 ± 11.65 vs –2.12 ± 9.96, *p* < 0.001: respectively). But the difference in the mean changes of social performance was borderline significant (7.16 ± 13.59 vs –0.96 ± 12.01, *p* = 0.05). The mean changes of other QOL parameters such as physical performance, physical limitations, and physical pain, were not significantly different between the two groups (*p* = 0.09, *p* = 0.14, and *p* = 0.06, respectively) (Table 5).

**Table 5:** The quality of life parameters at baseline and post-intervention.

Variables	Intervention group (*n* = 25)	Control group (*n* = 25)	*p*-value^**^	*p*-value***
Physical performance				
Baseline	66.00 ± 15.94	60.00 ± 22.77	0.28	
After 3 months	67.40 ± 14.72	58.60 ± 21.33	0.09	
*p*-value*	0.44	0.32		
Difference^b^	1.40 ± 8.95	−1.40 ± 7.00		0.09
Physical limitations				
Baseline	43.00 ± 42.40	33.00 ± 44.90	0.35^b^	
After 3 months	51.40 ± 36.38	33.69 ± 41.70	0.10^b^	
*p*-value^a^	0.23	0.31		
Difference^b^	8.40 ± 37.74	−2.17 ± 10.42		0.14
Emotional limitations				
Baseline	40.64 ± 45.16	30.46 ± 38.42	0.48^b^	
After 3 months	68.04 ± 35.38	22.00 ± 33.62	< 0.001^b^	
*p*-value*	0.006	0.14		
Difference^b^	27.40 ± 41.69	−8.64 ± 26.92		0.002
Energy and freshness				
Baseline	50.60 ± 11.93	54.40 ± 14.95	0.32	
After 3 months	66.60 ± 12.88	53.00 ± 15.20	0.001	
*p*-value*	< 0.001	0.09		
Difference^b^	16.00 ± 8.66	−1.40 ± 3.95		< 0.001
Mental health				
Baseline	52.16 ± 14.02	56.80 ± 13.80	0.24	
After 3 months	61.96 ± 10.63	55.68 ± 14.13	0.08	
*p*-value*	< 0.001	0.23		
Difference^b^	9.80 ± 8.92	−1.12 ± 4.54		< 0.001
Social performance				
Baseline	67.56 ± 19.45	59.24 ± 18.27	0.10^b^	
After 3 months	74.72 ± 12.17	58.28 ± 17.70	< 0.001^b^	
*p*-value^a^	0.02	0.80		
Difference^b^	7.16 ± 13.59	−0.96 ± 12.01		0.05
Physical pain				
Baseline	63.04 ± 15.63	64.84 ± 16.62	0.76^b^	
After 3 months	67.68 ± 11.47	62.48 ± 16.15	0.12^b^	
*p*-value^a^	0.07	0.44		
Difference	4.64 ± 15.05	−2.36 ± 13.38		0.06
General health				
Baseline	43.48 ± 10.46	49.04 ± 14.44	0.12	
After 3 months	61.48 ± 12.49	46.92 ± 14.74	< 0.001	
*p*-value*	< 0.001	0.29		
Difference	18.00 ± 11.65	−2.12 ± 9.96		< 0.001

Values are expressed as means ± SD. *p < 0.05 was considered as significant using Paired *t*-test. **p < 0.05 was considered as significant using Independent *t*-test between the two groups at baseline and post-intervention. ***p < 0.05 was considered as significant difference using Independent *t*-test between the two groups post-intervention. ^a^*p* < 0.05 was considered as significant using Wilcoxon test. ^b^*p* < 0.05 was considered as significant using Mann–Whitney U test between the two groups at baseline and post-intervention.

## Discussion

Oxidative stress has been suggested as a key factor in the onset of diabetes and its complications. According to studies, oxidative stress caused by hyperglycemia leads to oxidation of nucleic acids and proteins and develops cardiovascular complications in diabetic patients. So, antioxidants are very important for the treatment or prevention of the progression of diabetic cardiac complications (33, 34). To the best of our knowledge, the present study was the first well-designed clinical trial to evaluate the antioxidant properties of rutin in diabetic patients. The findings of this 3-month clinical trial showed that intervention with rutin resulted in a significant improvement in the status of antioxidant enzymes, blood pressure, and QOL in patients with T2DM. Similar to the results of this study, animal studies have shown that administration of rutin improved the antioxidant status in diabetic rats (35–38). Contrary to the present study, a clinical trial was conducted by Ramzy Ragheb et al. (2020) to evaluate the effects of vitamin C (160 mg) alone and in combination with rutin (60 mg) in patients with T2DM. The results of this 8-week study did not show a significant improvement of oxidative stress parameters, such as MDA and SOD (23). The different design and the lack of an independent intervention group for the rutin in this study, the use of a lower rutin dose, and a shorter intervention period can be the probable reasons for the difference in results between the two studies. In another study, inhibition of amylin-induced neurotoxicity and decreased production of ROS and MDA were observed with the use of rutin. So, the role of rutin in increasing the activity of the antioxidant enzymes, such as CAT, SOD, and GPx, and inhibiting amylin accumulation may be the reasons for the protective and antioxidant effects of rutin (39). One of the proposed mechanisms for the antioxidant effects of rutin is to prevent the activation of nuclear factor-κB (NF-κB) and reduce the production of inflammatory mediators, such as TNF-α, IL-6, mitogen-activated protein kinase (MAPK), cyclooxygenase-2 (COX-2), and inducible nitric oxide synthase (iNOS) (40). It has also been suggested that rutin may inhibit the biosynthesis of lipoxygenase (LOX) and cyclooxygenase (COX) and thus reduce the production of eicosanoids and inflammatory mediators (41).

On the other hand, blood pressure is one of the complications of diabetes that oxidative stress plays an important role in its development and progression (42). Mutually, the progression of hypertension and diabetes leads to endothelial dysfunction, a reduction of antioxidant enzymes activity, and an increase in oxidative stress and pro-inflammatory markers (43). Therefore, it seems that there is a two-way relationship between oxidative stress and blood pressure in diabetes, and the prevention of these two can help improve the treatment of diabetes and its complications. Consumption of beverages and foods containing flavonoids as well as supplements containing flavonoids can play a beneficial role in improving diabetes and its associated complications (44). Epidemiological studies have described activities, such as capillary strengthening and lowering blood pressure, for rutin antioxidant (22, 45). The results of this study also indicated that the use of rutin improved SBP, DBP, PP, MAP, and HR in T2DM patients. Some animal studies have confirmed the anti-hypertensive effects of rutin (46, 47). A study on diabetic rats with myocardial infarction reported that rutin consumption improved electrocardiogram (ECG) components (QT and QRS intervals) and HR and significantly reduced the infarct size. These special effects of rutin were accompanied by increased activity of CAT and SOD antioxidant enzymes (48). In addition to animal studies, the effects of rutin on lowering blood pressure have been demonstrated in human research. In line with this research, a clinical trial by Sattanathan et al. reported that rutin 2-month treatment significantly reduced SBP and DBP in patients with T2DM (27). However, in that study, there was no control group to compare the results with the intervention group (27). One of the proposed mechanisms for the antihypertensive effect of rutin is the stimulation of the nitric oxide/guanylate cyclase (NO/GC) pathway, which leads to vasodilation. It also acts as an antihypertensive agent by inhibiting acetylcholinesterase (ACE) and the angiotensin-converting enzyme (ACE) and inhibition of angiotensin II production (49).

It has been shown that diabetes and its complications affect the QOL of patients (50). Therefore, new perspectives have proposed the evaluation of QOL as a new indicator in assessing the clinical condition of diabetic patients (51). Previous reports have documented the effects of rutin and other treatments on improving QOL (13, 52, 53). In agreement with the earlier studies, the researchers in the current study found the effects of rutin on improving the mental dimension of QOL in patients with T2DM. Similar to the clinical findings of this study, Ragheb et al. reported that the use of a combination of vitamin C with rutin further improved QOL parameters (physical performance, physical limitations, energy and freshness, physical pain, emotional limitations, social performance, mental health, and general health) compared with vitamin C alone in diabetic patients. However, the use of vitamin C alone also had significant effects on QOL (23). In the present clinical trial, the positive effects of rutin were seen in improving the mental aspects of QOL in diabetic patients but rutin consumption did not show a significant increase in the physical symptoms of QOL. It may be argued that longer-term interventions (more than three months) with higher doseare needed to affect physical symptoms of QOL in diabetic patients (especially older people, who are more affected by the disease) and maybe a three-month period is not enough to improve the physical aspects. Considering the role of oxidative stress in diabetes, it can be said that by reducing oxidative stress and improving the complications of diabetes, the QOL also increases in these patients. However, this issue needs clarification.

A proper experimental design (having a control group and randomization along with allocation concealment) and a three-month intervention period were the strengths of this clinical trial. Also, in this study, modeling was performed and the effect of confounding factors (BMI, WC, sex, age, race, education, job, medications, physical activity, disease duration, and dietary intake) was controlled, which increased the accuracy of the results. In addition, due to the use of the ITT approach and analysis of results with a maximum sample size, the study power was increased. On the other hand, perhaps it can be said that in order to generalize the results to the general population and to recommend the use of rutin as adjunctive therapy, this sample size is small and is considered one of the weaknesses of the present study. The lack of a follow-up period after the end of the intervention to ensure that the effects of the intervention remain could be another limitation of the intervention.

## Conclusion

The results of this study supported the advantageous effects of rutin on blood pressure, antioxidant status, and mental parameters of QOL in diabetes. Therefore, it can be said that rutin may be a beneficial choice for patients with T2DM. However, to prove this claim, more clinical trial studies with larger intervention periods and large sample sizes are needed to generalize the finding to the general population.

## Data availability statement

The raw data supporting the conclusions of this article will be made available by the authors, without undue reservation.

## Ethics statement

The studies involving human participants were reviewed and approved by the protocol of this clinical trial was confirmed by the Ethics Committee of the Ahvaz Jundishapur University of Medical Science (IR.AJUMS.REC.1400.110). The patients/participants provided their written informed consent to participate in this study.

## Author contributions

HB and VA: all steps of research and writing the manuscript. AZ: supervision, all steps of research, and writing the manuscript. AA, SH, SY, and MG: drafting of the manuscript. FZ: critical revision of the manuscript for important intellectual content. VZ: drafting and revision of the manuscript. All authors reviewed the manuscript and approved the final version of the manuscript.

## Funding

The study was financially supported by the Student Research Committee of Ahvaz Jundishapur University of Medical Sciences (project number: 00s13).

## Conflict of interest

The authors declare that the research was conducted in the absence of any commercial or financial relationships that could be construed as a potential conflict of interest.

## Publisher’s note

All claims expressed in this article are solely those of the authors and do not necessarily represent those of their affiliated organizations, or those of the publisher, the editors and the reviewers. Any product that may be evaluated in this article, or claim that may be made by its manufacturer, is not guaranteed or endorsed by the publisher.

## Abbreviations

BMI: Body mass index
WC: Waist circumference
HC: Hip circumference
SBP: Systolic blood pressure
MAP: Mean arterial pressure
DBP: Diastolic blood pressure
HR: Heart rate
PP: Pulse pressure
SOD: Superoxide dismutase
GPx: Glutathione peroxidase
CAT: Catalase
T2DM: type 2 diabetes mellitus
ELISA: Enzyme linked immunosorbent assay
